# Public Engagement to Inform Messaging Aligning With the Muscle‐Strengthening Guidelines

**DOI:** 10.1002/gin2.70083

**Published:** 2026-08-10

**Authors:** Ashley Gluchowski

**Affiliations:** ^1^ School of Health & Society University of Salford Salford UK

**Keywords:** adults, communication, exercise, promotion, public health, resistance

## Abstract

**Introduction:**

Physical activity guidelines and their supplementary messaging play a fundamental role in raising awareness and increasing knowledge at a population level. In the United Kingdom, there are no national‐level messaging campaigns accompanying the strength component of their physical activity guidelines for public health. Perhaps unsurprisingly, then, research indicates a very low awareness of and adherence to the strength component of these guidelines.

**Methods:**

This study used the Physical Activity Messaging Framework (PAMF) during a participatory workshop to inform messaging guidance aimed to accompany the UK's Chief Medical Officers' muscle‐strengthening guidelines. We focused on a subgroup of middle‐aged women. Twenty adults (*n* = 18 females) aged 40–60 years residing in the Greater Manchester area participated in one, 4‐h, in‐person workshop. Participants were in the contemplation, preparation, action, maintenance, or termination stage of the Transtheoretical Model of behaviour change. The focus group activities and resulting discussions aligned with the PAMF to identify message content, format, and delivery that resonated with the target audience.

**Results and Discussion:**

Discussions revealed that it is simply not enough to tell people to do muscle‐strengthening exercise (even when including information about frequency, muscle groups, or intensity) if we hope to increase self‐efficacy, competence, or change behaviour. Individuals need more details on *how* to practically participate in muscle‐strengthening exercise. Those who do not know how or who do not feel confident they know enough, simply will not start, revealing a clear enactment barrier. As a solution, participants proposed that future messaging campaigns include links to more detailed information on *how* to participate in muscle‐strengthening exercise, practically and effectively. This is the first study to use the PAMF to develop messaging recommendations for (inter)national‐level mass media campaigns aligned to the muscle‐strengthening guidelines.

## Summary

- **What Is Already Known on the Subject.**
  ∘ Evidence suggests that as little as 4% of adults adhere to the muscle‐strengthening exercise guidelines.
  ∘ Mass media messaging campaigns are recommended to encourage higher levels of awareness and participation in physical activity guidelines.
  ∘ However, the physical activity guidelines currently lack an evidence‐based messaging strategy for muscle‐strengthening exercise.
- **What This Study Adds.**
  ∘ This study presents 11 initial recommendations for muscle‐strengthening exercise messaging campaigns as a result of public engagement with key stakeholders.
- **Implications for Practice, Research, and Policy.**
  ∘ The UK's House of Commons, Health and Social Care Committee report on ‘Healthy Ageing: Physical Activity in an Ageing Society’ has recommended a ‘national movement campaign’ focusing on ‘examples of movement that will increase strength and balance’ – this research takes the first step towards developing consistent, relevant, and salient muscle‐strengthening exercise messages for future national‐level movement campaigns.

**Abbreviations**
ACSM: American College of Sports Medicine
PAMC: Physical Activity Messaging Checklist
PAMF: Physical Activity Messaging Framework
PAMF/C: Physical Activity Messaging Framework and Checklist
QR: Quick Response
WHO: World Health Organization

## 1 Introduction

Globally, 31.3% of adults do *not* adhere to the aerobic component of international physical activity guidelines [1]. This is in stark contrast to the 22.8% of adults who *are* reported to be meeting the muscle‐strengthening component of those same guidelines [2]. Other reports suggest that as little as 7.3% of men and just 4.1% of women engage in the recommended levels of muscle‐strengthening activity [3]. Besides being female, individuals of an older age, individuals clinically categorized as overweight and obese, and those with poor self‐reported health are even less likely to adhere to the muscle‐strengthening guidelines [2, 3, 4]. Moreover, unlike aerobic activity, there are methodological challenges in capturing the prevalence of participation and adherence to muscle‐strengthening activity [5]. Brummer et al. found that while 71.1% of participants reported participating in the recommended frequency for muscle‐strengthening activity, only 26.8% of those participants met the frequency, intensity, *and* muscle group recommendations [5]. To date, we must rely on cross‐sectional self‐report, in‐depth interview, ethnographic observation, or, perhaps, more ideally, long‐term objective muscle strength measurement. Each has severe limitations to the feasibility or accuracy of our findings and conclusions.

Regardless, there continue to be many barriers facing the awareness, adoption, and adherence to muscle‐strengthening activity, especially when compared to aerobic activity. Notably, the barriers to muscle‐strengthening activity include the (real and/or perceived) need for specialized equipment, facilities, and knowledge and skills [6, 7, 8]. It is no surprise, then, that muscle‐strengthening activity as an exercise modality and the (inter)national guidelines promoting muscle‐strengthening exercise do not seem to resonate well with the public; muscle‐strengthening activity is still perceived to be relevant only for those looking to achieve a ‘muscular’ appearance or to improve sports performance [8, 9, 10]. Current guidelines are also seen to be lacking clarity, offering little information on ‘how’ to practically get started with muscle‐strengthening exercise [10, 11]. Taken together, these barriers may be creating psychological resistance to starting muscle‐strengthening activity, especially among insufficiently active or sedentary subgroups.

The obvious lack of a messaging strategy, especially when compared to its aerobic counterpart, has likely contributed to dangerously low levels of muscle‐strengthening participation [12]. However, both the World Health Organization (WHO) and the International Society for Physical Activity and Health (ISPAH) recommend the use of mass media campaigns with clear, consistent messages to encourage higher levels of awareness and participation in physical activity [13]. And while there are several media campaigns in the United Kingdom targeting physical activity (for girls: www.thisgirlcan.co.uk; and for people living with long‐term conditions: www.weareundeatable.co.uk and www.csp.org.uk/campaigns-influencing/campaigns/stronger-my-way), to date, few studies have co‐designed messages with distinct target audiences to improve the dissemination of the muscle‐strengthening guidelines. To our knowledge, there are no national‐level campaigns that specifically address muscle‐strengthening exercise upstream, that is, for younger or middle‐aged adults without long‐term health conditions or multimorbidity. Most recently, the UK's House of Commons, Health and Social Care Committee report on ‘Healthy Ageing: Physical Activity in an Ageing Society’ has recommended a ‘national movement campaign’ focusing ‘on examples of movement that will increase strength and balance’ [12]. However, taking a preventative approach represents a key opportunity to build strength and robust healthy lifestyle habits as one prepares to enter later life [14, 15]. Therefore, using a participatory approach, this study aims to provide initial recommendations for future work looking to align strength‐based messaging with the national physical activity guidelines, emphasizing the voice of middle‐aged women. ‘Participatory approach’ was operationally defined as a process where participants acted as ‘co‐researchers’ in messaging design during an interactive, bidirectional workshop and not simply taking part in a one‐way data collection process. Using the Physical Activity Messaging Framework and Checklist (PAMF/C) as a guiding framework, the results of this study, when implemented, could help to influence muscle‐strengthening activity awareness, knowledge, motivation, intention, and, with time, behavioural outcomes and social norms [16, 17, 18].

## 2 Methods

This study took a qualitative approach, utilizing focus groups to explore the naturalistic insights of participants who would collectively have the relevant experience to explore the research aim [19]. Participants with relevant experience were identified through an online, mixed‐methods survey that was released in December 2023 (onlinesurveys.co.uk) and advertised on social media. One hundred and nineteen people responded to the online survey before it closed in January 2024. We purposively invited 20 of those 119 responders who also met the inclusion criteria (residing in the geographical region of Greater Manchester, England, and who indicated on their survey that they would be willing to participate in one, in‐person, half‐day workshop). We also emphasized middle‐aged women and purposively invited those across the range of stages in the transtheoretical model of behaviour change (apart from precontemplation). Purposive sampling prioritizing behavioural stage led to a diverse cohort in the stages of behaviour change, but did not achieve diversity in ethnicity (see Table 1).

**Table 1:** Self‐reported participant demographics (*n* = 20).

Category	Subgroup	*n*
Sex	Female	18
	Male	2
Age	40–49	10
	50–59	8
	60+	2
Ethnicity	British	17
	Irish	1
	Asian	1
	Caribbean	1
Education	Master's	7
	Bachelor's	11
	High school	2
Employment	Full time	13
	Part time	5
	Stay at home	1
	Retired	1
Transtheoretical Model stage of change	Precontemplation	0
	Contemplation	3
	Preparation	2
	Action^a^	8
	Maintenance	6
	Termination	1

^a^ Action stage included participants who reportedly participated in muscle‐strengthening exercise, but participation was reported to be irregular, infrequent, underdosed (once per week), or twice per week for less than 1 year.

In qualitative research, saturation is typically reached with 9–17 interviews and 4–8 focus groups [20]. Twenty participants in four focus groups participated in this research. See Table 1 for self‐reported participant demographics. The reporting of this study conforms to the items within the COREQ checklist [21].

### 2.1 Focus Groups – Behavioural Segmentation

Twenty participants were assigned to one of four focus groups (*n* = 5 in each focus group). Participants were assigned to numbered focus groups by their Transtheoretical Model stage of change. Group 1 contained participants who had not participated in muscle‐strengthening exercise previously (i.e., participants self‐reported to be in the contemplation or preparation stages of change). Group 2 contained participants who had some limited experience in participating in muscle‐strengthening exercise (i.e., participants were taking irregular action or in the relapse/termination stage). Group 3 contained participants currently participating in a once‐per‐week muscle‐strengthening exercise programme (underdosed action stage). Finally, Group 4 contained participants who were consistently participating in muscle‐strengthening exercise at least twice per week (the recommended dose) for over a year (i.e., participants were in the maintenance stage) [22]. Therefore, each focus group displayed behavioural homogeneity and thus, participants were able to share social experiences with the topic. Overall, the workshop displayed heterogeneity, allowing the author and her ‘co‐researchers’ to explore and contrast diverse perspectives around ‘ideal’ strength messaging.

### 2.2 PAMF

Focus group activities were closely aligned with the PAMF/C developed by Williamson et al. Decisions that were pre‐specified included ‘I am using this checklist to create a new message’, ‘target population identified’, ‘target audience engaged with to inform message development’, ‘time of year’, ‘specific aim of the message,’ ‘how the message is intended to work,’ and ‘why the decisions around message creation being made’ (see Table 2). All other categories of the PAMF/C are the result of iterative, bidirectional discussions and written statements among the 20 co‐researchers.

**Table 2:** Pre‐determined decisions aligned to the Physical Activity Messaging Checklist (PAMC) [16].

	Results
I am using this checklist to create a new message	Yes, a new strength‐based physical activity message
Who? (in which groups?)	Pre‐specified decisions
Target population identified	Males and Females, 40–60 years old, living in the Greater Manchester region, United Kingdom.
Target audience engaged with to inform message development	Yes, 20 participants were involved and informed of these messaging recommendations via in‐person focus groups. Males (*n* = 2) and Females (*n* = 18), 40–60 years old, living in the Greater Manchester region, United Kingdom.
When? (in which context?)	Pre‐specified decisions
Time of year and social/political context of the message considered	Workshop took place in January 2024, potentially capitalizing on the new year motivation.
What? (Is the aim?)	Pre‐specified decisions
Specific aim of the message is identified, and specific outcomes relating to the aim are clearly stated	Aim of the message is to
	− improve knowledge surrounding muscle‐strengthening exercise (targeting competence) [23] − improve motivation to participate in muscle‐strengthening exercise (targeting motivation) [23]
	Outcomes of interest include
	− influencing current messengers/campaigns to include muscle‐strengthening exercise in their physical activity messages (see 11 recommendations in Section 5, below) − produce campaign poster prototype (with the ability to be tested, refined, and disseminated in the future – both printed and online) (see Figshare link)
How? (Is it to work?)	Pre‐specified decisions
Potential pathway(s) by which the message may bring about change in the outcome(s) of interest identified (e.g., targeting beliefs about capabilities)	Providing co‐produced evidence directly to current messengers/campaigns on what the targeted cohort finds relevant and motivating with regard to muscle‐strengthening messaging.
	Directly increasing attention and awareness, and attempting to influence competence and motivation by
	− visual reminders alongside nudges/cues to action (via links to practical knowledge on ‘how’ to practically participate in strength exercise) − repeated exposure to upward social comparison via social modelling/norms
Why?	Pre‐specified decisions
Decisions based on co‐production with the target audience	Yes, decisions based on engagement with the target audience in January 2024 (one‐time, 4‐h, in‐person workshop)
Decisions based on existing literature and evidence involving the target audience	Yes, decisions to complete this piece of research were based on existing literature suggesting a lack of awareness and participation in muscle‐strengthening exercise [10]

### 2.3 Researcher Reflexivity

The author (female, PhD, University Fellow and Clinical Exercise Physiologist, and experienced qualitative workshop facilitator) reports a bias for, and an interest in, effective ways to support evidence‐based muscle‐strengthening exercise participation and adherence among middle‐aged and older adults. She has academic and clinical knowledge and experience in interviewing and focus groups with the public around muscle‐strengthening exercise awareness and (barriers to) participation. During this research, she attempted to act as a ‘passive’ moderator, that is, to keep the discussion on time, on topic, and exploratory rather than steering the conversation. No relationship was established with participants prior to the workshop, but participants were aware (via the ethics information sheet) of the reasons for the research. No non‐participants were present, and no participants dropped out of this study.

### 2.4 Analysis

Qualitative data from focus group activities were transcribed by each group using paper and pens provided. Paper transcriptions were translated by the author into electronic documents verbatim and coded using NVivo 12 (qrsinternational.com). Participants were offered £75 as a token of appreciation following the workshop. Utilizing the PAMF/C and its pre‐determined categories as semantic themes, data was co‐constructed in a thematic manner and reported in a pragmatic way, appropriate for public and governmental consumption and future implementation [24, 25, 26]. Representative quotations are presented verbatim.

Specifically, in addition to the author facilitating the workshop, and thus being immersed in the day's discussions, she submerged herself in the written data from each group, transcribing and then reading and re‐reading all transcripts in the weeks following the workshop. Coding was systematic and thorough, moving forward and backward through familiarization and coding, identifying patterns across the dataset that were relevant to the study's aim [24, 25, 26]. Phases 3–5 in the thematic analysis method (theme development, refinement, and naming) were pre‐determined by the PAMF/C categories set forth by Williamson et al. [16].

The findings were taken back to four participants (one participant from each group) as an additional opportunity for co‐constructed understanding and collaborative reflection. Their reflections are presented at the end of Section Results and Discussion, 3.

## 3 Results

The workshop began and ended with the same question [19, 27]. Prior to any discussion, participants were asked ‘what is the first thing you think of when I say muscle‐strengthening exercise?’ Participants wrote their answer on paper, anonymously (except for indicating their group number), and then placed it in an envelope marked ‘before’. Participants were asked the same question at the end of the 4‐h workshop. This time, they were also asked to put either ‘(same)’ or ‘(different)’ on their responses to indicate whether their thoughts and associations had stayed the same or had changed. Again, responses were anonymous to discourage social desirability bias. As such, ‘before’ and ‘after’ responses cannot be matched to each other and are separated only by focus group number (see Table 3, below).

**Table 3:** ‘Muscle‐strengthening exercise’ association results (presented verbatim).

Group	Before workshop commences	After workshop concludes
1	Weights	Functional fitness (different)
	Lifting weights	Necessary (different)
	Squats	Improved health (different)
	Body building	Less dementia (different)
	Support for ageing	Squats/weights exercises to help strengthen for health (different)
2	Weight training	Resistance training (different)
	Compound barbell exercises	Important (different)
	Hard work	Varied (different)
	Boring	Longevity (different)
	Something I enjoy and am actively wanting to return to doing regularly	Something that needs to become the norm (different)
3	Weights	Essential (different)
	Exercising	Achievement (different)
	Power	Fitness (different)
	Pain	Health (different)
	Getting shredded	Progression (different)
4	Weights	For everyone (different)
	Lifting weights	Good health (different)
	Resistance loading	Health (different)
	Instagram	Resistance (same), Feel good factor (different)
	World's strongest man	World's strongest man (same)

The subsequent themes and subthemes are in direct alignment with the Physical Activity Messaging Checklist and as a result of focus group questions, iterative, bidirectional activities, and resulting collaborative discussions [16].

### 3.1 Theme – Type of Information

#### 3.1.1 Subtheme – Message Contains ‘What to Do’ Information

As a starting point, we asked participants what they liked and disliked about three major physical activity guideline infographics: the UK's Chief Medical Officers' physical activity guidelines, the WHO's guidelines on physical activity and sedentary behaviour, and the American College of Sports Medicine's resistance training for health.

Participants liked that the UK's Chief Medical Officers put the strength guideline ‘in the middle’ of their infographic. The infographic predominantly includes frequency guidance, which was seen as ‘simple’, ‘helpful’, and ‘achievable’. The examples of modalities include carrying heavy bags, yoga, and the gym. Inclusion of some modalities appeared to confuse participants, ‘is yoga muscle‐strengthening exercise?’ and ‘Yoga is not muscle‐strengthening exercise’. Furthermore, participants required additional information to act – that is, beyond frequency, participants thought the strength guideline ‘lacks clarity on specifics (e.g., time to aim for)’, and ‘duration and intensity not addressed’, making this infographic ‘not specific enough’, ‘vague, too general’ and Group 3 simply stated ‘But how?’

Participants liked the images on the WHO's infographic, as they were seen as ‘better icons’, ‘more representative’ and although outlines, appeared to be a ‘normal person’ unlike the ‘blob’ figures in the UK's infographic. The more realistic icons showing ‘physical action’ gave the impression that strength could be ‘done be anyone at a park bench’. However, participants thought the wording in this infographic was ‘repetitive’ and many questions were raised, ‘what are the chronic conditions?’ ‘what are major muscle groups?’ and ‘what is moderate or greater intensity?’ Possibly pointing to the need for fuller definitions or explanations to be listed or linked to.

When asked to look at the American College of Sports Medicine resistance training for health infographic (listing repetitions, sets, tempo, intensity, etc.), participants stated, ‘we all really like this, it gives clear examples’, and ‘very informative and specific’. Thus, this infographic was seen as sufficiently detailed and most likely to overcome the enactment barrier. On the other hand, the cohort thought there was enough information on this infographic to potentially be split into two infographics to further increase readability, as it is ‘very wordy but does tell you everything’.

Overall, participants agreed that as a solution, future guidelines and accompanying messaging and infographics should include more details directly or include a quick response (QR) code linking to more detailed plans/programmes to further improve the ‘what to do’ message.

#### 3.1.2 Subtheme – Message Contains ‘Why You Should Do It’ Information

Participants felt muscle‐strengthening exercise participation should be promoted with slogans along the lines of ‘be the best you’ and ‘keep doing what you love’, showing an innate preference for more autonomous motives. Additionally, participants felt the focus of future messaging should (i) ‘add mental health benefits’, (ii) include physical function, and (iii) include physical health benefits.

Both short and long‐term benefits were of interest to the cohort. Participants wanted quick wins and were also motivated by longer‐term factors such as delaying the onset or slowing the progression of age‐related cognitive conditions, ‘[messaging should] mention, keep away dementia’.

There was still a deeply ingrained misconception that muscle‐strengthening exercise was *solely* for appearances, for those who wanted to look ‘muscular’, or for those looking to improve their sports performance. Participants urged any future messaging to avoid feeding into this narrative.

#### 3.1.3 Subtheme – Message Contains ‘How to Do It’ Information

Participants felt that the ‘how’ to practically *do* muscle‐strengthening exercise was the most important component for any guideline and associated messaging.

Participants wanted exercise examples and demonstrations, tips on when and how to progress their training, access to both online and in‐person provisions, ‘real life role models sharing their stories’, ‘at both ends of the strength spectrum’ as well as ample signposting to all these options.

Progressive overload (an important strengthtraining principle) was a surprise to most everyone, even those who were already participating in the recommended frequency of muscle‐strengthening exercise. Some regularly exercising participants admitted to doing ‘one set of the same exercises for over a year’ or simply ‘going through the motions’.

Relating to the lack of knowledge and use of progressive overload, participants noted that only the very basic movements and programming variables were currently available on their ‘go‐to’ websites for health and wellness information (trusted, national‐level websites; e.g., www.nhs.uk). Participants wanted future messaging guidance and provision to be more ability‐inclusive by including options for beginner, intermediate, and advanced/robust abilities and participants, stating options should be available ‘for whatever your level’.

The preferred framing and targeting of muscle‐strengthening exercise messaging can be found in Table 4, below.

**Table 4:** The framing and targeting of messaging.

THEME Framing, targeting	Results via in‐person focus group discussions
*Subtheme*	
Message content is gain‐framed	Yes, muscle‐strengthening exercise messaging should emphasize the benefits of participation
Message content is loss‐framed	No, loss‐framed messages were not used or preferred
Message content is generic (suitable for all)	Somewhat; participants particularly liked the generic message, ‘muscle‐strengthening exercise is recommended and beneficial for all ages and abilities’.
Message content is tailored to an *individual*	No, not feasible in mass media messaging
Message content is *targeted* at a group	Yes, targeted to an age (40–60 years) and geographical (Greater Manchester) demographic. Content is also targeted at adults between the contemplation and termination stage of change in the transtheoretical model of behavioural segmentation. As such, this (targeted but not tailored) messaging will likely *not* directly influence those in the precontemplation stage.
Message content is *personalized*	No, not feasible in mass media messaging

### 3.2 Theme – Use of Language

#### 3.2.1 Subthemes – Appropriate Language and Choice of Words Considered

When discussing the use of appropriate language and choice of words within muscle‐strengthening messaging campaigns, some strong objections to certain terms were evident. For example, there was a preference for ‘risk reduction’ over ‘prevention of disease’ for some participants (especially from Group 1) as ‘prevention’ could be seen as ‘victim blaming’. Additionally, the terms ‘elderly’, ‘senior’, and ‘old’ were deemed inappropriate.

Participants felt it was important to emphasize inclusive messaging, such as ‘everyone has their own starting point’ and that the ‘benefits of participation outweigh the risks’.

#### 3.2.2 Subtheme – Message Conveyed Using a Particular Tone

Tones used in messaging should be age‐positive, such as ‘getting better’ as you get older, inclusive, and encouraging, both individually and culturally. Encouraging a ‘culture’ of muscle‐strengthening exercise participation was a key solution discussed.

The preferred content and format of muscle‐strengthening exercise messaging can be found in Table 5, below.

**Table 5:** The content and format of muscle‐strengthening exercise messaging.

Theme Message content	Results via in‐person focus group discussions
*Subtheme*	
Message uses text to convey information	Great use of text included:
	− personal success stories in the form of quotations − detailed information on programming (repetitions, sets, intensity, progression)
Message uses images or videos to convey information	Photographs/images of a variety of real, age‐appropriate people (and even of older (> 60 years old) than the targeted demographic) with different body types were all seen to be most inspiring. Images of those who were younger than the targeted cohort (< 40 years old) did not seem to have the same inspiring effect.
	Participants wanted to see sweaty people, being ‘active and strong in a variety of ways’ (home, park/outdoors, work/office, gym/exercise class).
	Cartoons of people were not preferred (stick/blob icons were the least preferred).
Message uses music	No, music did not appear to be important or helpful.

THEME Message format	Results via in‐person focus group discussions
*Subtheme*	
Radio advert	Briefly mentioned, not preferred format
TV advert	Briefly mentioned, not preferred format (if used, display during the evenings and weekends)
Poster	Predominately poster based, with a QR code to link to further information (especially, *how* to practically *do* muscle‐strengthening exercise)
Leaflet or pamphlet	Not mentioned; preference for electronic options (app‐based)
Social media post	Platforms discussed include Instagram and Facebook, with little mention of TikTok, Snapchat, and Twitter
	Participants use social media in the evenings
Email	Briefly mentioned, possibly as nudges, every 6 months
SMS/text message	Not mentioned
Other	Workplace computer screens were seen as an important yet missed opportunity to display messages
The message is of a specified length or volume	Mixed; both short and long‐form content were appreciated and should be used in collaboration (short‐form content that allows users to easily access more detailed information on demand)

### 3.3 Theme – Message Delivery

#### 3.3.1 Subtheme – Healthcare Professional (HCP)

Participants acknowledged that their HCPs were not likely to give muscle‐strengthening exercise guidance; however, messages on the bulletin boards and TV screens in clinical waiting rooms were seen as an effective setting where people could read/learn more independently.

#### 3.3.2 Subtheme – Peers

Peers appeared to be the preferred main source of message delivery. Participants wanted to see success stories from peers (‘real life’ people they could identify with), especially peers who have successfully managed to overcome barriers, make muscle‐strengthening exercise a weekly habit, and have seen health and/or wellness benefits.

#### 3.3.3 Subtheme – Celebrities

Celebrities were not mentioned. However, older (> 60 years old) real‐life people who are successfully participating in muscle‐strengthening exercise and are featured or are trending on social media (‘fitness influencers’) were seen as inspiring and motivating.

#### 3.3.4 Subtheme – Others

The employer/workplace was seen as a major missed opportunity. Participants felt that muscle‐strengthening messages, guidance, and provision should be promoted and delivered during the workday.

Overall, participants wanted to see consistent messages (and branding) regardless of who delivers them.

### 3.4 Theme – The Setting in Which the Message Will Be Delivered Has Been Considered and Specified

Again, the employer and the workplace were seen as a *major* setting in which we should be delivering messages and provision, due to time spent in the workplace, the importance of staying economically active in later life (a current issue in the United Kingdom), and muscle‐strengthening exercise's perceived fit within existing health and wellbeing packages. Transport spaces (e.g., at bus stops, or on trains) were also mentioned as highly visible spaces where messaging would be seen.

Gyms were noted as especially relevant, as even though participants may be aerobically active, they likely needed more information on how to effectively participate and adhere to muscle‐strengthening exercise.

### 3.5 Theme – What Can We Learn From Physical Activity Campaigns?

Participants were shown a number of current physical activity campaigns. In the campaign by the Chartered Society of Physiotherapy (CSP), ‘stronger my way’, images of real, normal‐looking people in their home were used [28]. Participants felt there was good use of story telling and a QR code that linked to more information. Participants presumed provision to be evidence‐based, as they would expect from a professional body.

Participants in the current study felt that the ‘this girl can’ campaign was a well‐known, (body) positive campaign, and they enjoyed that the campaign showed girls working hard [29]. The current cohort (aged 40–60 years) found the perceived target age range of this campaign, and the use of the word ‘girl’ unrelatable. Participants felt there was no perceived direction on ‘what to do’ or ‘how to do it’, so they were unsure what the call to action was. Importantly, as a female‐based campaign, participants in the current study wanted to see (more) mention and information surrounding menopause.

The campaign ‘we are undefeatable’ targets people living with long‐term health conditions and was not seen as inspiring as some of the other campaigns (even to the non‐muscle‐strengthening exercise focus group, some with self‐reported health conditions) [30]. Participants felt that the models used in the campaign could have looked more active and happier. The ‘5 in 5’ tagline (for a 5‐min workout) was easy to remember. Participants noted that it was not (overtly) clear how trustworthy (or evidence‐based) the campaign was.

‘Couch to fitness’ is a free web‐based fitness programme, and was seen to have a clever name (albeit ‘fitness’ was seen as a little vague) because of the positive association with the well‐known ‘couch to 5k’ aerobic exercise programme in the United Kingdom [31]. Participants appreciated the honest quote on their poster campaign (‘I found the gym daunting’), but perceived this programme to be targeting a younger cohort due to their images. Participants would have liked to have seen a success story and more information about what component of ‘fitness’ this programme was targeting. The importance of easy and obvious linking to more information was stressed. Again, participants were not clear whether this was an evidence‐based programme.

Participants felt the CrossFit campaign rode a fine line between inspiring and age‐positive, and the perception of ‘cultish’ or elitist [32]. Participants wanted CrossFit to make it clear that beginners are welcome. CrossFit could also have more effectively capitalized on the health and social benefits of participation. A greater diversity in their images would also be appreciated.

### 3.6 Collaborative Reflection

These findings were taken back to four participants (one from each focus group) who were asked to share their reflections on the written findings. This step acted as an additional opportunity for the researcher and her co‐researchers to work together and ensure the co‐constructed interpretation continued to represent and resonate with lived experiences.

Two participants strongly reiterated the significance of the theme ‘use of language’ and how the words we choose can shape attitudes, can help challenge (or perpetuate) stereotypes, and ultimately influence how people feel about ageing and the behaviours they take (or not). One participant was particularly ‘pleased that the report highlighted the use of terminology and the importance of using appropriate, respectful language. In particular, the acknowledgement that terms such as “elderly,” “senior,” and “old” are considered inappropriate is something I feel strongly about…’ Another reflected ‘inclusive and appropriate wording of messaging still holds really true, framed in a positive way rather than seeking to blame/shame’.

One participant noted that although the findings highlight a generic message appropriate for mass media messaging (‘muscle‐strengthening exercise is recommended and beneficial for all ages and abilities’ in Table 4), upon reflection, she noted, ‘do we in fact need to dial it up/differentiate it when required? e.g. beneficial for all, but particularly for certain groups – e.g. women in mid‐life, adults approaching retirement’.

Other areas that appeared to particularly resonate with participants included the ‘why you should do it’ subtheme, as the knowledge of the cognitive benefits of muscle strength and muscle‐strengthening participation acted as a ‘reality check’ for one participants' own behaviour. Within the theme ‘message delivery’, peers were indeed especially relevant in delivering messages, noting that future messaging should include ‘people that we could identify with, that even despite barriers you could find ways to fit muscle‐strengthening exercise into your life’.

Interestingly, one participant reflected that ‘ever since doing this piece of research, I'd say there's much more publicity about the importance of muscle‐strengthening exercise … I would say still missing from this is good education/info such as the importance of progressive overload – e.g., lots of videos of women doing bicep curls with tiny 1 kg dumbbells. Do we need to focus more messaging about this now??’.

Finally, all four participants found that even after the workshop, the findings ‘still hold true’, and ‘still feel relevant and current’, while one noted that these findings may be even more important now, especially in light of the ‘House of Commons focusing on muscle strengthening messaging as this could make such a vital difference to so many people's wellbeing and lives’.

## 4 Discussion

Utilizing the PAMF/C [16], we have provided initial recommendations for messaging and campaign teams looking to improve the awareness, knowledge, and participation in the muscle‐strengthening guidelines [33]. Participants in this study were in the contemplation, preparation, action, maintenance, and termination stages of behaviour change and were quite obviously interested in the topic of muscle‐strengthening exercise. Results show that there was a strong preference for ‘how to do it’ content, evident throughout the day's discussions and resulting quotations. It was clear that ‘how’ to perform muscle‐strengthening exercise (practically and effectively) was the main barrier to uptake and regular, appropriately dosed participation. For example, participants were unsure what exercises, what load, or what number of repetitions to complete. Thus, we would encourage future messaging to redirect the focus from ‘what to do’ and ‘why to do it’ – giving ‘how to do it’ greater precedence. Participants urged future mass media campaigns to prominently feature QR codes linking directly to practical, evidence‐based muscle‐strengthening exercise information, such as videos created by qualified muscle‐strengthening exercise professionals. This additional information would help to overcome enactment barriers and facilitate help‐seeking behaviours by educating this cohort on the principles of safe and effective muscle‐strengthening exercise participation. Of note, participants resonated particularly well with the infographic from the American College of Sports Medicine as it provided a clear and detailed training plan (‘perform 2–3 sets of 8–12 repetitions with good form, lift and lower the weight in a controlled manner…’), which other guidelines and campaigns made less obvious or lacked altogether [34].

### 4.1 Comparison With Literature

To the best of the author's knowledge, muscle‐strengthening exercise has not been the focus of the PAMF, messaging or science communication research, or mass media campaigns to date. In fact, it has been recommended that more care should be given to provide the public with clear, direct, and consistent muscle‐strengthening messaging [12, 33]. The lack of focus on muscle‐strengthening exercise is shown in Stead et al. In their comprehensive review of public health messaging literature, they found 36 papers, 7 of which included physical activity. Those seven reviews targeted brisk walking, time spent walking, stair use, changes in physical activity, and self‐reported activity, but none included muscle‐strengthening activity [35]. However, parallels can be drawn from the current study's findings to a scoping review of 123 studies of general physical activity messaging [18]. Williamson et al. found that physical activity messages targeting adults should be framed positively, include short‐term mental health benefits, be delivered through peers, and use real images [18, 36, 37]. There is also support for the use of practical ‘how to’ advice in general physical activity messaging [36, 37].

Our findings are also similar to an online slogan experiment conducted in Australia [38]. Pettigrew et al. tested different physical activity slogans in adults aged 50 years and older and found that in the youngest group (50–59 years), adults were most motivated by messages providing quantitative, evidence‐based, and explicit guidance (‘be active 30–60 minutes a day to stay fit and well’) [38]. A preference for explicit guidance seems to mirror other reports in Greater Manchester residents [39]. There does appear to be differences between adults and older adults. Adults in older groups (60–69 and 70+ years) seem to prefer more generic slogans without a quantitative threshold. Older cohorts may also expect physical activity messaging to come from their health care providers [18]. Interestingly, there were no differences in slogan preferences between males and females, an important finding since the current study contained mostly female participants [38].

### 4.2 Limitations

There are important limitations in the current study. The voluntary nature of research means that people who have an inherent interest in the topic engaged and responded to our initial survey. While physical activity research is unlikely to engage those in the precontemplation stage, our purposive sampling did draw across all other stages of the transtheoretical model of behaviour change (contemplation, preparation, action, maintenance, termination). The half day, in‐person, and participatory aspects of this study did limit the number of participants that could feasibility and meaningfully participate in this research. The presence of a facilitator, however passive, may still provide subtle cues that may influence group dynamics or discussion content. In addition, the responses were captured by participants themselves; while empowering, this could have also led to missing nuance that an audio‐recorded transcript may or may not have captured. Using the PAMF/C, although evidence‐based, introduces the possibility of reduced depth or the ability to capture emergent themes outside predetermined PAMF/C categories. However, the discussion around physical activity guideline infographics and other messaging campaigns did allow for inductive analysis. As with most qualitative or public engagement studies, the sample was small. However, the study did reach a sample of middle‐aged females in full‐time work, a cohort highly unlikely to be participating in sufficient or effective levels of muscle‐strengthening exercise [2, 3]. Thus, these insights may be key to improving the prevalence of muscle‐strengthening activity in this cohort.

On the other hand, the cohort was educated, a consistent facilitator to adhering to healthy behaviours, including the physical activity guidelines. All participants were living in Greater Manchester, in the United Kingdom. Given the low global participation rates, especially in women of middle‐age, findings may translate to other geographical regions. However, we were unable to achieve ethnic diversity. Due to the demographic characteristic of our sample, findings may not be directly transferable to men, younger adults, or people in the pre‐contemplation stage and caution should be used before these findings are translated to these cohorts.

As this study is the first to use participatory approaches to provide recommendations for muscle‐strengthening messaging campaigns in an attempt to improve awareness, knowledge, and participation in muscle‐strengthening exercise, it is suggested that future research replicate this work to strengthen these results while targeting more diverse cohorts. Future research could also undertake individual interviews to delve deeper into specific themes and findings to draw out further nuance. Resultant messaging should also be evaluated prior to implementation at scale, including theoretically plausible ways in which messaging is expected to bring about changes in behaviour (e.g., by improving the competence and motivation components of the COM‐B) [23].

The practice‐oriented, participatory approach underpinned by the consensus‐derived PAMF are notable strengths of this research. This study has allowed for an initial exploration of this cohort's preferences for ‘ideal’ muscle‐strengthening messaging. There was consensus amongst the focus groups and thus, consensus amongst people of various stages of behaviour change (contemplation through to termination).

### 4.3 Implications for Messaging Development

The results of this study may provide a vast number of organizations with the evidence they need to provide more relevant muscle‐strengthening exercise messaging, including but not limited to other academics, the National Health Service (NHS), national and local governments and authorities, mass media/social media marketing teams, and physical activity and/or ageing charities and consensus and guideline drafting committees. Importantly, this evidence begins to address the recent ‘Healthy Ageing: physical activity in an ageing society’ report by the UK's House of Commons Health and Social Care Committee – where it was recommended that a ‘national movement campaign’ include targeted messaging focusing ‘on examples of movement that will increase strength and balance’ [12].

Developing consistent, relevant, and salient muscle‐strengthening messaging may help to influence awareness, inform people on guideline targets, signpost to opportunities, provide inspiration and motivation, impact attitudes and social norms, and in time, increase desirable health behaviours [33, 40]. We present *11 recommendations for muscle‐strengthening exercise messaging campaigns:*

1. Use *images* of real people being active and strong in a variety of ways, focusing on diversity of body types, ethnicities, and ages. Avoid stick people or cartoon icons.
2. Quotes and *success stories* are an excellent use of text.
3. *Peers* are the preferred source of information and inspiration. Capitalize by asking more people to share their stories and experiences (see guidelines 1 and 2).
4. Even people who are planning to start or who are already participating believed that muscle‐strengthening exercise was only for physical appearance or sports performance. Including other *benefits* may be key to moving more people along to the next stage of change and for long‐term adherence (from contemplation to preparation or from action to maintenance).
5. Both *short‐term* benefits (like blood glucose regulation) and *long‐term* risk reduction (for instance, dementia) were effective and should be used simultaneously.
6. People self‐reporting to be participating in muscle‐strengthening exercise are admittedly ‘going through the motions’ or are quite inconsistent in their approach (as a direct result of not seeing any tangible benefits from their initial efforts), and were further encouraged with information about the importance of *progressive overload* for continued benefits and longer‐lasting results.
7. Messages should be clear and *consistent* across messengers (to avoid confusion and frustration), but also need to be seen across several channels and settings. Employers and workplaces were believed to play a major role in promoting and encouraging strength‐based exercise.
8. Effective messages are quickly and easily *linked* to more information and opportunities, preferably via QR code linking to a mobile app or dedicated web page.
9. Inclusive messages include information and opportunities for all ages and *all abilities*, beginner to advanced.
10. Information coming from recognizable, *credible* sources were more effective (professional exercise bodies, relevant charities, and universities).
11. Finally, but most importantly, it is not enough to tell people to do muscle‐strengthening exercise (even when including frequency, muscle groups, or intensity). People need more details to know *how* to *practically* participate in muscle‐strengthening exercise. People who do not know how or who do not feel confident they know enough, simply will not start.

These 11 recommendations provide the foundational data required for future empirical testing and refinement, especially in more diverse subgroups (such as lower socioeconomic status populations).

## 5 Conclusions

Mass media messaging is one of the ‘eight investments that work’ to influence health seeking behaviour, uptake, and life‐long adherence to the physical activity guidelines for the social, mental, and physical health and wellbeing of our society [13]. It is our hope that these 11 recommendations, designed with the public, can help when planning and promoting future muscle‐strengthening messaging campaigns.

## Author Contributions

**Ashley Gluchowski:** conceptualization, investigation, funding acquisition, writing – original draft, methodology, validation, visualization, writing – review and editing, software, formal analysis, project administration, data curation, resources.

## Ethics Statement

This study was reviewed and approved by the University of Salford's Research Ethics Board (2023‐0073‐41). Participants were sent the information sheet and consent form via email, and written informed consent was obtained prior to the in‐person workshop. The study was conducted in accordance with the Declaration of Helsinki.

## Conflicts of Interest

The author declares no conflicts of interest.

## Data Availability

Metadata underpinning this publication are openly available from the University of Salford Figshare at https://doi.org/10.17866/rd.salford.28624757.
